# Targeting beta-2 adrenergic receptor attenuates schizophrenia-like behavioral effects induced by ketamine in mice: cAMP/PKA/BDNF-PEA-3 and RIM-1α signaling pathways involvement

**DOI:** 10.1007/s44446-026-00079-x

**Published:** 2026-04-29

**Authors:** Mohammed M. Heikal, Ahmed F. Mohamed, Nora O. Abdel Rasheed, Dalia M. El-Tanbouly, Noha N. Nassar

**Affiliations:** 1 Postgraduate Program in Pharmacology and Toxicology, Faculty of Pharmacy, Cairo University, Cairo, Egypt; 2 Pharmacology and Toxicology Department, Faculty of Pharmacy, Cairo University, Kasr El-Aini St., Cairo, 11562 Egypt; 3 Faculty of Pharmacy, King Salman International University (KSIU), South Sinai, 46612 Egypt; 4 Pharmacology and Toxicology Department, October University for Modern Science and Arts (MSA), Giza, Egypt

**Keywords:** Schizophrenia, Ketamine, Formoterol, CAMP/PKA/BDNF, Neuro-inflammation, Synaptic plasticity

## Abstract

The interplay between cAMP/PKA and neuroplasticity involves intricate signaling pathways that are crucial for brain function and are frequently disrupted in schizophrenia. This study sought to examine the potential neuroprotective effects of formoterol on ketamine-induced schizophrenia-like behaviors with emphasis on several PKA downstream crucial targets for schizophrenia management. Male mice were injected with ketamine (20 mg/kg/day, i.p) for 14 days. From day 8, animals were treated with formoterol (100 μg/kg) with or without, the PKA inhibitor, H89 (0.05 mg/kg). Behavioral endpoints were assessed with n = 15 per group, and following sacrifice animals were stratified into subsets for downstream analyses: histopathology (n = 3), biochemical/neurochemical assays (n = 6), and molecular profiling including western blotting (n = 3) and qRT‑PCR (n = 6). Formoterol improved ketamine- induced anxiety, impaired social interaction and anhedonic behavior. It also restored neurochemical balance and enhanced learning and memory functions. Formoterol attenuated neuro-inflammation and oxidative stress, and modulated synaptic plasticity. Formoterol-induced neuroprotection could be attributed to its boosting action on cAMP/PKA/BDNF signaling to promote RIM-1α and PEA-3 gene expression. Consequently, it upregulated glutamate NMDA receptor subunits namely; GluN2A, and GluN2B and augmented expression of vital synaptic plasticity regulators; kalirin-7, PSD-95, synaptophysin and synapsin-2. Accordingly, formoterol is a promising candidate against schizophrenia-associated synaptic dysfunction and neurotransmitters imbalance. Formoterol neuroprotective effects were abolished upon administration of PKA inhibitor confirming that the cAMP/PKA cascade is a vital key-player in the drug favorable effects.

**Supplementary Information:**

The online version contains supplementary material available at 10.1007/s44446-026-00079-x.

## Introduction

Schizophrenia is a severely impairing mental disorder recognized by both positive and negative symptoms, as well as social and cognitive impairments. Positive symptoms including delusions, hallucinations, disorganized speech, and catatonic behavior besides negative symptoms, such as alogia, avolition, and affective flattening were recorded as common symptoms of schizophrenia (Larson et al. 2010). The lifetime prevalence of schizophrenia ranges from 0.30% to 0.66% (McGrath et al. 2008), and affected individuals typically have a lifespan that is 12 to 15 years shorter than the average, a disparity that continues to widen (Saha et al. 2007). Neuroplasticity, which is the capacity of the brain to restructure itself through the creation of new neural connections, has a vital role in understanding the pathophysiology as well as the treatment of schizophrenia. Research has shown that schizophrenia is associated with impaired neuroplasticity, particularly in the motor cortex and prefrontal regions (Bhandari et al. 2016; Exner et al. 2006; Radhu et al. 2015; Rubia et al. 2001; Webler et al. 2020). Deficits in synaptic plasticity—such as reduced dendritic spine density, altered expression of synaptic scaffolding proteins, and impaired long-term potentiation (LTP)—have been implicated in the cognitive and negative symptoms of schizophrenia (Wu et al. 2022).

The 3', 5’-cyclic adenosine monophosphate/protein kinase-A (cAMP/PKA) cascade has been linked to various neuro-psychiatric disorders emphasizing its role in maintaining proper neuronal function (Semesta et al. 2023). Similarly, cAMP/PKA signaling is an intriguing cascade that is implicated in schizophrenia pathogenesis (Tardito et al. 2000). Activation of cAMP provoked PKA expression with subsequent phosphorylation of the nuclear transcription factor cAMP response-element binding protein (CREB) leading to consequent activation of the brain-derived neurotrophic factor (BDNF) which regulates survival, synaptic plasticity, and long-term memory (Weis and Kobilka 2018). Phosphorylation of CREB is also implicated in the enhancement of N-methyl-D-aspartic acid receptor (NMDAR)-mediated synaptic responses in addition to improved memory, learning and neural plasticity (Brigman et al. 2010). Thus, cAMP/PKA/pCREB signaling cascade is deemed crucial for NMDA receptor functioning (Wang and Peng 2016).

Additionally, Rab3-interacting molecule- 1α (RIM-1α) is a downstream target of PKA which plays a role in triggering presynaptic long-term potentiation (LTP) which is a type of activity-dependent plasticity which improves synaptic transmission. LTP requires pre- and post-synaptic depolarization that is accomplished via the NMDARs (Lonart et al. 2003). While postsynaptic LTP has a broader distribution, presynaptic LTP occurs in some of the brain's most prevalent synapses, such as those in the hippocampus and corticostriatal regions (Garcia-Junco-Clemente et al. 2005). Besides, kalirin-7, the predominant isoform of kalirin expressed in the adult brain, is essential for spine development and synaptic plasticity. Thus, in addition to spine loss and reduced glutamatergic transmission in the frontal cortex, kalirin-knockout mice also show impairments in working memory, social behavior, and increased locomotor activity that are linked to schizophrenia. These were reversed by the atypical antipsychotic clozapine (Cahill et al. 2009). Postsynaptic density 95 (PSD-95), a key protein in the postsynaptic membrane, functions as a link between NMDARs and downstream signaling molecules, facilitating synapse maturation. Research has demonstrated that increased expression of PSD-95 in hippocampal neurons promotes glutamatergic synapses maturation and boosts glutamate receptor activity, thereby supporting synaptic plasticity and stabilization (Taft and Turrigiano 2014).

Ketamine, a noncompetitive NMDAR antagonist, temporarily induces symptoms and cognitive deficits that mimic schizophrenia manifestations (Javitt et al. 2012; Stone et al. 2008). Previous research has showed that the administration of ketamine in mice reproduces numerous behavioral and neurochemical characteristics associated with schizophrenia. Behavioral symptoms observed include hyperactivity, stereotyped behavior, social and cognitive impairments (Ben-Azu et al. 2018; Jeevakumar et al. 2015). The neurochemical and molecular alterations encompass changes in neurotransmitters such as Ach, reduced expression of GABAergic neurons, and oxidative stress, which are implicated in the pathophysiology of schizophrenia (Fujikawa et al. 2021; Monte et al. 2013; Schiavone et al. 2020). Therefore, ketamine has been widely utilized as a representative animal model for studying schizophrenia (Bubeníková-Valešová et al. 2008).

Formoterol, a prolonged-acting and selective agonist of β2 adrenoceptors (β2AR) (Anderson 1993), is commonly used to alleviate respiratory complications linked to chronic pulmonary diseases and asthma, and is characterized by its rapid onset and prolonged duration of action (LaForce et al. 2005). Notably, a study by Dang et al. showed that formoterol enhances synaptic plasticity and improves cognitive function, likely by targeting central β2ARs, as the drug is capable of crossing the blood–brain barrier (Dang et al. 2014). Activation of β2AR is known to modulate intracellular cAMP levels, thereby influencing the PKA/CREB/BDNF axis and promoting neuroplasticity (Gupta et al. 2025). Moreover, adrenergic signaling has been implicated in the regulation of neuroinflammatory responses, which are increasingly recognized as contributors to schizophrenia pathophysiology (Chaves et al. 2024). Elevated levels of pro-inflammatory cytokines such as TNF-α and IL-6 have been reported in patients with schizophrenia, and β2AR stimulation has been shown to attenuate such inflammatory markers (Peterson et al. 2025). In Parkinson's disease, formoterol has been noted to enhance mitochondrial dynamics by reducing oxidative stress, thereby offering neuroprotective benefits (Chang et al. 2024). These findings suggest that β2AR agonists may exert dual therapeutic effects, enhancing synaptic plasticity and mitigating neuroinflammation, both of which are relevant to schizophrenia.

Given the limited efficacy of existing antipsychotics for cognitive and negative symptoms and the central role of synaptic plasticity deficits in these domains, this study tested whether β2AR activation by formoterol can reverse ketamine-induced schizophrenia-like phenotypes via engagement of the cAMP/PKA pathway and downstream plasticity regulators (BDNF-related signaling, RIM-1α, and PEA-3).

## Materials and methods

### Experimental procedures

#### Drugs and chemicals

Formoterol was obtained from Novartis (Basel, Switzerland) and ketamine was obtained from EIPICO (Cairo, Egypt, B.N: 2,101,183). Both KT and formoterol were prepared in normal saline (Yang et al. 2010). The PKA inhibitor, H-89 “dihydrochloride hydrate” was obtained from Sigma (St. Louis, MO, USA). H89 was dissolved in 1% DMSO (Seyedi et al. 2014). Other chemicals used in the study were of the highest analytical grade.

#### Animals

Adult Swiss albino male mice (*n* = 75), each weighing 20–25 g, were sourced from the National Research Center. The animals were kept under regulated temperature conditions (22 ± 2 °C), with humidity maintained at 60% ± 15%, and a regular 12-h light/dark cycle with ad libitum access to food and water. Cage maintenance was performed twice weekly, excluding testing days. All animal care and handling procedures adhered to the US National Institutes of Health's (NIH) Guide for the Care and Use of Laboratory Animals (publication No. 85–23, 2011 revision) and were conducted under the regulations of the Animal Experimentation Ethics Committee of the Faculty of Pharmacy, Cairo University, Egypt (approval No. PT8995-11/2020). Following adaptation, the mice were divided into 5 groups, each comprising 15 mice, as detailed in the "Experimental design".

### Experiment design

Mice were assigned to 5 groups, each consisting of 15 mice, at random. Group I: received 0.9% saline for 14 days to serve as the normal control group. Group II: was administered 0.9% saline for 7 days then on the 8th day treatment was initiated on formoterol (100 μg/kg, i.p) daily for another 7 days (Abdel Rasheed et al. 2018). Group III: received a daily i.p. injection of ketamine (20 mg/kg) for 14 days, serving as the ketamine control group (Vasconcelos et al. 2015). Group IV: was administered a daily i.p. injection of ketamine (20 mg/kg) for 14 days and formoterol (100 μg/kg, i.p) every day for 7 days, starting from the 8th day, 30 min after the ketamine administration. Group V: received a daily i.p. injection of ketamine (20 mg/kg) for 14 days and H-89 (0.05 mg/kg, i.p.) one hour before formoterol (100 μg/kg, i.p.) every day for 7 days, starting from the 8th day (Seyedi et al. 2014).

**Fig. 1:**
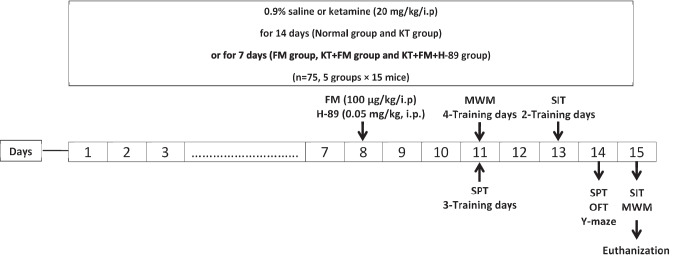
Experimental design

All drug administrations were performed during the light phase of the circadian cycle, between 09:00 and 10:00 AM, to minimize variability due to hormonal or metabolic fluctuations. Behavioral testing was conducted at 11:00 AM as demonstrated in the experimental timeline. (Fig. 1). Consistent timing across days and groups was maintained to ensure reproducibility and reduce confounding circadian effects. Behavioral scoring was conducted by observers blinded to treatment allocation, and animals used for biochemical and molecular assays were randomly selected from those subjected to behavioral testing.

### Behavior assessments

#### Open field test (OFT)

Locomotor activity was assessed using the open field test (Kraeuter et al. 2019). This assessment was conducted to ensure that the outcomes of the behavioral tests were not attributable to alterations in the mice's locomotor activity but to schizophrenia-associated anxiety. A square-shaped white acrylic open box with a white bottom and brown edges, each side measuring 95 × 95 cm and 50 cm high, was used (Cunha and Masur 1978). A red marker was used to divide the white bottom into sixteen identical squares. Each mouse was separately positioned in the middle of the arena and observed for a duration of 3 min to assess the following metrics:

1. Latency: which refers to the duration (in seconds) from the moment the animal is placed at the center of the box to its initiation of movement (Zbinden 1981).
2. The frequency of ambulation: which denotes the total count of squares traversed in a minute. The total count of squares traversed in the 3-min observation time was utilized in group comparisons (Stanford 2007).
3. The frequency of grooming: which captures the frequency of the animal's face wash, scratch, and fur licking within the three-minute observation period (Chow and Beck 1984).
4. The frequency of rearing: which measures the number of instances the mouse stood upright on its back legs and stretched, within the observation period (Altman et al. 1971).

#### Y-maze spontaneous alternation

This test assesses short-term memory (Luszczki et al. 2005). In the present study, a wooden Y-maze consisting of 3 arms in the form of a Y shape was employed. The arm measurements are as follows: 40 cm in length, 30 cm in height, and 15 cm in width, positioned 120° apart from the center. Typically, normal mice exhibit a tendency to investigate a novel arm rather than a previously encountered one. The arm entries sequence for each mouse was recorded during the 8-min period. To eliminate olfactory cues that could lead to inaccurate observations, the compartments were cleaned using 75% ethanol following each mouse test. An actual alternation refers to consecutive entries into all three arms, forming overlapping triplet sets. Possible alternations are calculated by subtracting 2 from the total count of arm entries. The spontaneous alternation percentage is calculated by dividing actual alternations by possible alternations and multiplying the result by 100 (Yamada et al. 1999). Only those mice that demonstrated eight or more arm entries were included in the analysis of spontaneous alternation behavior.

$${\% Alternation}=\left(\frac{Number of actual alternations}{Total arm entries-2}\right)\times 100$$

#### Social interaction test (SIT)

The social interaction test's general layout was modified from (File 1980). The test was conducted in a neutral cage made of clear Plexiglas (25 cm × 20 cm × 20 cm) without a top. On days 1 and 2, each mouse underwent a 10-min adaptation session each day, during which it was placed alone in the neutral cage to acclimate to the apparatus. On day 3 (test day), each mouse was paired with an unfamiliar mouse in the cage for 10 min. The unfamiliar mouse was a sex- and age-matched male that had no prior contact with the test subject. These mice were housed in separate cages under identical environmental conditions and were habituated to handling but not to the testing apparatus. Each unfamiliar mouse was used only once per day to avoid repeated exposure effects and was randomly assigned to test subjects to minimize bias. Behavior was video-recorded using a camera positioned 80 cm above the cage. The time of social interaction, including active behaviors like sniffing, grooming, following, mounting, and crawling over or under the partner, was measured over the 10-min period. Passive contact, such as sitting or lying with bodies touching, was not included as part of social interaction.

#### Sucrose preference test (SPT)

This test is employed to assess anhedonia in mice which is a negative symptom of schizophrenia (Zhou et al. 2011). To assess sucrose preference, mice were individually housed. The protocol consisted of three phases: (1) Adaptation: On day one, mice were continuously subjected to two graduated drinking tubes for 72 h, one had tap water and the other a solution of 2% sucrose. All mice had free access to lab chow. (2) Deprivation: started directly after adaptation, mice were deprived of both food and water for 20 h. (3) Test: following deprivation, all groups were subjected to 3-h access to one bottle of tap water and one bottle of a 2% sucrose solution. The sucrose preference was calculated as the percentage of sucrose consumption relative to the total drinking volume.

$$Preference =(\frac{Sucrose intake}{Total intake}) \times 100\%$$

#### Morris water maze (MWM) test

The aim of the test is to assess the animals’ learning abilities and their visuospatial memory (Rolls 2000). A metallic cylinder-shaped tank (180 cm in diameter and 60 cm in height) was utilized in this study. The pool was half-filled with water maintained at room temperature and divided into four equal quadrants using two threads fixed perpendicular to each other at the rim of the pool. A small, black-painted Plexiglas escape platform (10 cm in diameter) was placed inside the target quadrant, 2 cm below the water surface. The position of the platform remained the same during the procedure. To make the water non-transparent, a non-toxic dye with a purple hue was applied, rendering the platform indistinct. Animals rapidly learn to swim straight toward the platform under normal circumstances, which allows them to arrive there faster. The assessment was conducted over 5 consecutive days (Gupta and Gupta 2012). Each animal underwent 2 consecutive trials on the four test days, with a minimum of fifteen minutes separating each trial. Each trial had a maximum duration of 2 min. If the animal located the platform within 2 min, it remained on it for 20 more seconds before removal. Mice that failed to locate the platform within the designated timeframe were gently led to it and allowed to remain there for 20 s. As an index of learning or acquisition, the mean escape latency (MEL) time, representing the duration each animal took to locate the hidden platform, was documented for every trial throughout the four testing days (Singh et al. 2013). A probe trial session was conducted on the fifth day, during which the platform was taken out of the pool and each mouse was given sixty seconds to investigate the pool. As a measure of memory retrieval, the time each mouse spent in the target quadrant, previously containing the hidden platform, was measured (Morris 1984).

### Tissue sampling

Upon conclusion of the behavior tests, mice were decapitated by cervical dislocation under intraperitoneal sodium thiopental anesthesia (Reilly 2001). Following euthanization, mice were stratified into three analytical subsets, each designated for distinct histological, biochemical, molecular, and proteomic investigations. Whole brains were carefully excised and immediately fixed in 10% neutral-buffered formalin. These samples were processed for histological examination employing hematoxylin and eosin (H&E) staining (subset 1, n = 3).

Bilateral hippocampi were dissected, washed with ice-cold saline, and homogenized in 10% saline using a Heidolph Diax 900 homogenizer. The homogenate was centrifuged at 15,000 rpm for 25 min at 4 °C to obtain the supernatant. This supernatant was then employed for detailed biochemical (cAMP, PKA, TNF-α, IL-1β, caspase-3, MDA, NO, H₂O₂, GSH) and neurochemical profiling (Ach, GABA, glutamate, BDNF, PSD-95, synaptophysin) via ELISA and colorimetric assays (subset 2, n = 6).

Hippocampal tissues (subset 3, n = 6) were subdivided for molecular and proteomic analyses. For Western blotting (n = 3), right hippocampi were homogenized in RIPA lysis buffer using a Heidolph Diax 900 homogenizer, centrifuged at 15,000 rpm for 25 min at 4 °C, and the resulting supernatant was subjected to SDS-PAGE and immunoblotting to assess synaptic protein expression (kalirin-7 and synapsin-2). For qRT-PCR (n = 6), total RNA was extracted from three whole hippocampi and three left hemispheres. Transcript levels were quantified for genes implicated in synaptic modulation (RIM-1α, PEA-3) and receptor signaling (GluN2A and GluN2B).

### Biochemical and neurochemical parameters determination

GSH, MDA, glutamate, and Ach levels were quantified utilizing ELISA kits (MyBioSource, CA, USA, cat# MBS724815, MBS741034, MBS2601720 and ELK Biotechnology, TX, USA, cat# ELK8593, respectively). ELISA kits were also employed to estimate the levels of TNF-α, IL-1β, and caspase-3 (Cusabio, Wuhan, China, cat# CSB-E04741m, CSB-E08054m and CSB-E08858m, respectively). These parameters were determined following the manufacturer's instructions for each ELISA kit and were expressed in units corresponding to the tissue protein content as determined by the method of Lowry et al. (1951). BDNF and cAMP were determined using Creative Diagnostics ELISA kit, NY, USA (cat# DEIA712 and DEIA2964, respectively). Color absorbance was determined at a wavelength range of 490 to 630 nm. PKA, PSD95, and synaptophysin levels were measured using ELISA kits (LSBio, MA, USA, cat# LS-F28575 and LS-F7142, respectively) and (Elabscience, USA, cat# E-EL-M1105).

H_2_O_2_ and NO levels were measured using colorimetric kits (Enzo Life Sciences, New York, USA, cat# ADI-907–015 and Elabscience, USA, cat# E-BC-K035-S, respectively). GABA level was determined using a fluorometric assay kit (ELK Biotechnology, TX, USA, cat# ELK8591).

### Quantitative Real-time Polymerase Chain Reaction (qRT-PCR)

The gene expression of RIM-1α, PEA-3, GluN2A, and GluN2B were assessed via qRT-PCR. Tissue samples were processed in lysis buffer to create a homogenate, and total RNA was extracted using the SV Total RNA Isolation system (Thermo Scientific, USA). The extracted RNA was converted into cDNA using the high-capacity cDNA reverse transcription kit (#K4374966, Thermo Fisher Scientific, USA), following the manufacturer's instructions for the reverse transcription master mix preparation. Subsequently, qRT-PCR was carried out using SYBR Green I on an Applied Biosystem equipped with software version 3.1 (StepOne™, USA). The cDNA amplification employed the following primer sets: RIM-1α: forward, 5′-CTTCACCGGGTAGCGAGCCAGG-3′ and reverse, 5′-ATCCGAAAGGTGAGAGCCAGAGC-3′; PEA-3: forward, 5′-AGGAGACGTGGCTCGCTGA-3′ and reverse, 5′-AACCTAGCTTTCCACAGCCCC-3′; GluN2A: forward, 5′-ACATCCACGTTCTTCCAGTTTGG-3′ and reverse, 5′-GACATGCCAGTCATAGTCCTGC-3′; GluN2B: forward, 5′-CCAGAGTGAGATGGATTGC-3′ and reverse, 5′-TGGGCTCAGGGATGAAACTGT-3′. All cDNA samples, including previously prepared samples, internal controls (for β-Actin gene expression as the housekeeping gene), and non-template controls (to confirm the absence of DNA contamination), were run in duplicate. The thermal cycling conditions comprised an initial activation step at 50 °C for 2 min, followed by 40 cycles of denaturation at 95 °C for 15 s and a combined annealing/extension step at 60 °C for 60 s. The data were expressed in terms of Cycle threshold (Ct) values using the 2 − ΔΔCt method.

### Western blotting

Kalirin-7 and synapsin 2 were measured by a Western blot assay. Prior to transferring the protein bands onto cellulose acetate membranes, protein extracts from hippocampus tissue were separated using 10% sodium dodecyl sulfate–polyacrylamide gel electrophoresis (SDS-PAGE). The membranes were then incubated overnight at 4 °C with primary antibodies, specifically kalirin polyclonal antibody (Thermo Fisher Scientific, OH, USA, Cat. No. PA5-36,953; dilution 1:1000) and anti-synapsin-2 antibody (Cell Signaling Technology, MA, USA, Cat. No. 2312; dilution 1:1000). Following 3 washes with 0.1% TBST for 5 min each, the membranes were incubated with an HRP-labeled secondary antibody (goat anti-rabbit-IgG, HRP/IgG Goat mAb, Novus Biologicals, NB7187; dilution 1:1000) for 2 h at room temperature. Subsequently, chemiluminescent enhanced substrates (Clarity™ Western ECL substrate, BIO/RAD, USA, Catalog # 170–5060) were applied according to the manufacturer's instructions. Band intensities were quantified using the Chemi Doc MP imager after normalization to β-actin.

### Histopathological studies

The brains of three mice from each group were preserved in 10% formalin saline for 24 h. Following fixation, the tissues were washed, then dehydrated using a series of alcohol dilutions (methyl, ethyl, and absolute ethyl). Specimens were treated in xylene and encased in paraffin at 56 °C in a hot air oven for 24 h. Paraffin-embedded tissue blocks were then sectioned (4 μm thick) using a sliding microtome. The sections were mounted on glass slides, deparaffinized, and stained with hematoxylin and eosin (H&E) for examination of hippocampus as well as the prefrontal cortex under a light microscope (Bancroft and Gamble 2008).

### Statistical analysis

All data obtained were expressed as mean (x̄) ± SD. Using Shapiro–Wilk and Brown-Forsythe test, all results were examined for normality and homogeneity of variance, respectively. The MEL in Morris water maze trials was analyzed using repeated measures analysis of variance (ANOVA). The remaining results were analyzed using one-way ANOVA followed by Tukey's multiple comparison test. Statistical analysis was conducted using GraphPad Prism© software (version 6; Graph Pad Software, California, USA). For all statistical tests, a P-value of less than 0.05 was considered statistically significant.

## Results

### Effect of formoterol on the spontaneous locomotor activity of ketamine-injected mice

**Fig. 2:**
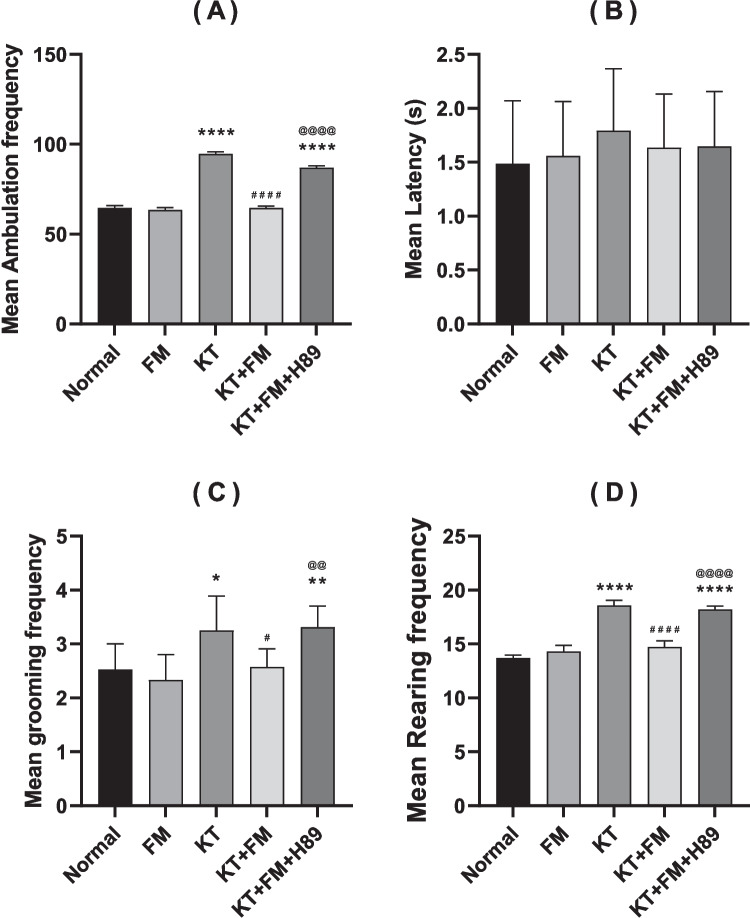
Effect of formoterol (FM) on spontaneous locomotor activity in ketamine-injected mice. (**A**) Mean ambulation frequency, (**B**) mean latency, (**C**) mean grooming frequency, and (**D**) mean rearing frequency. Values are expressed as mean ± SD; n = 15. ^*^P < 0.05, ^**^P < 0.01, ^****^P < 0.0001 vs normal group, ^#^P < 0.05, ^####^P < 0.0001 vs ketamine (KT) group, ^@@^P < 0.01, ^@@@@^P < 0. 0001 vs treatment group (KT + FM)

Ambulation, as well as grooming and rearing frequencies, were notably higher in the ketamine group— reaching 1.5, 1.3 and 1.4 folds, respectively as compared to the normal group, suggesting increased anxiety. Formoterol administration significantly reduced ambulation, grooming and rearing frequencies by 32%, 21.6%, and 21%, respectively, (P < 0.0001) as compared to ketamine group. No significant difference between the PKA-inhibitor group and the non-treated ketamine group was observed. Moreover, PKA inhibitor administration diminished formoterol–induced improvement in the formerly mentioned parameters by 35%, 30%, and 24%, respectively. Additionally, no significant alteration was recorded between the normal group and the formoterol-only group. No notable difference in latency was observed among the experimental groups. Therefore, the behavioral disturbances in the ketamine group can be attributed to ketamine-induced anxiety rather than locomotor deficits (Fig. 2).

### The effect of formoterol on ketamine-injected mice's spontaneous alteration behavior in the Y maze test

**Fig. 3:**
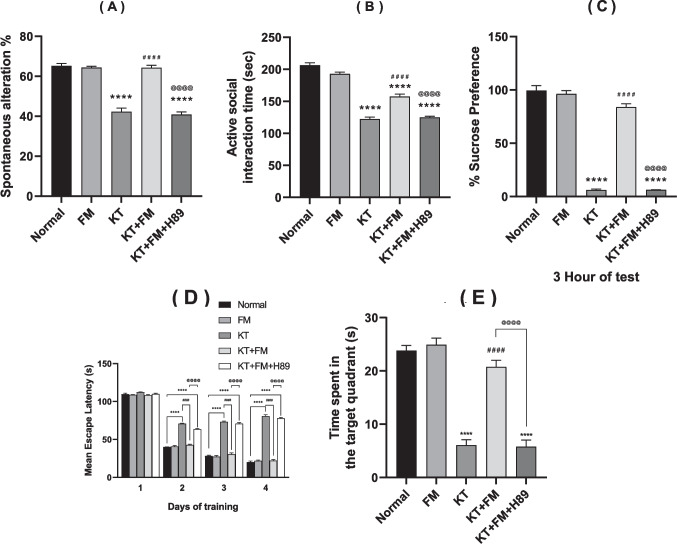
Effect of formoterol on spontaneous alteration behavior in Y maze task (**A**), social interaction test (**B**), sucrose preference test (**C**), Morris water maze mean escape latency (**D**) and the time spent in the target quadrant (**E**) in ketamine-injected mice. Values are expressed as mean ± SD; n = 15. ^****^P < 0.0001 vs normal group, ^####^P < 0.0001 vs ketamine (KT) group, ^@@@@^P < 0. 0001 vs treatment group (KT + FM)

Animals injected with ketamine demonstrated an obvious reduction in the percentage of spontaneous alteration behavior upon comparison with the normal group by 35%. Administration of formoterol led to a noticeably higher rate of spontaneous alteration behavior reaching 1.5 fold in comparison to the ketamine group (P < 0.0001). Administration of PKA inhibitor attenuated formoterol favorable effects on short term memory showing prominent reduction in spontaneous alteration behavior percentage by 36% which is equivalent to untreated ketamine group (Fig. 3A).

### The effect of formoterol on ketamine-injected mice's social interaction

Ketamine administration significantly reduced the duration of active social interaction by 41% (P < 0.0001) as compared to normal animals. However, treatment with formoterol markedly improved animals’ social interaction by 1.3 fold of the untreated-group, yet upon the PKA inhibitor administration, social withdrawal resembled that detected in ketamine-injected animals with prominent reduction by 20% as compared to formoterol treated animals (Fig. 3B).

### The effect of formoterol on ketamine-injected mice's sucrose preference

Mice injected with ketamine displayed a significant reduction in sucrose preference by 94% (P < 0.0001) on the test-day. Conversely, formoterol-treated mice demonstrated a significant improvement in sucrose preference reaching 13 fold (6.25 vs 84%). Animals that received the PKA inhibitor showed 92.6% decline in sucrose preference as compared to formoterol treated group which confirms PKA role in formoterol anti-anhedonic effect. Additionally, there were no differences in the total drinking volume among the groups (Fig. 3C).

### The effect of formoterol on ketamine-injected mice's MEL and time-spent in the target quadrant during the Morris water maze test

For every group, the average of the two trials that were carried out every training day was recorded. All groups exhibited MEL of 120 s. to find the platform on the first training day. Mice in every group arrived at the platform faster starting on the second training day, with the mice receiving ketamine injections showing the highest MEL. On days 2, 3, and 4, mice treated with formoterol displayed noticeably lower MEL than the ketamine group, whereas the MEL of mice injected with ketamine increased notably as compared to normal animals. Hence, formoterol treatment notably improved the ketamine-injected mice’s MEL on the four-training days (Fig. 3D). When compared to normal mice, the ketamine group showed a significant reduction in the amount of time spent in the target quadrant by 74.6%, where the platform was previously positioned, on the day of the probe test. Comparing to the ketamine group, the formoterol-treated group demonstrated a significant increase in the amount of time spent in the target quadrant, reaching 3.4 fold (P < 0.0001). Thus, administration of formoterol to ketamine group resulted in a significant improvement in spatial memory yet this positive impact was abolished upon the PKA inhibitor (H89) administration with significant reduction in time spent in the target quadrant by 72.2% as compared to formoterol treated group (Fig. 3E).

### The effect of formoterol on ketamine-injected mice's oxidative stress

**Fig. 4:**
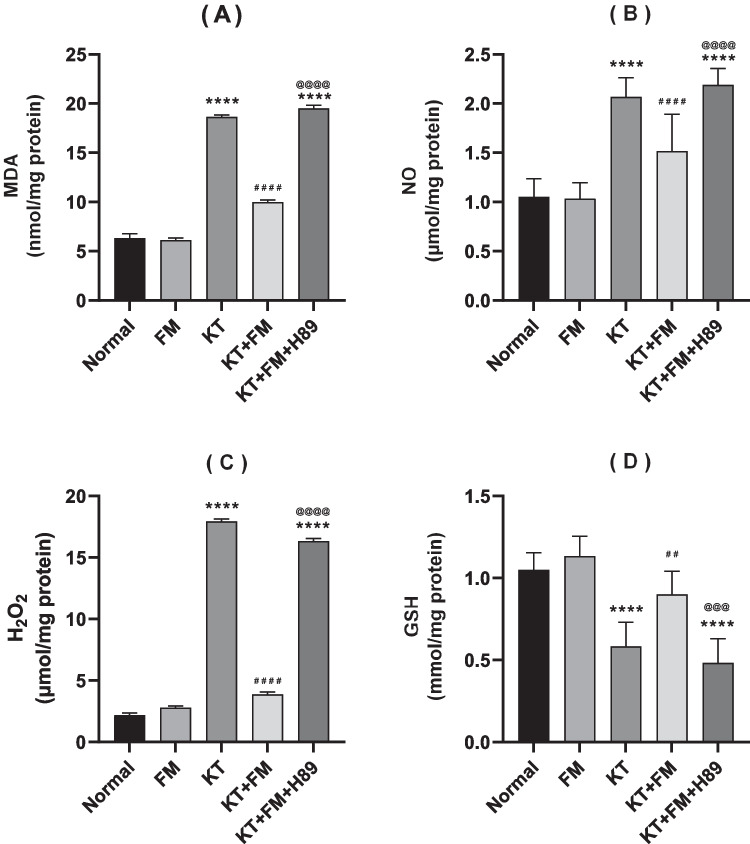
Effect of formoterol on ketamine-induced oxidative stress in bilateral hippocampal homogenates of mice. (**A**) MDA, (**B**) NO, (**C**) H_2_O_2_ and (**D**) GSH. Values are expressed as mean ± SD; n = 6. ^****^P < 0.0001 vs normal group, ^##^P < 0.01, ^####^P < 0.0001 vs ketamine (KT) group, ^@@@^P < 0.001, ^@@@@^P < 0. 0001 vs treatment group (KT + FM)

Ketamine group exhibited a notable increase in MDA, NO, and H_2_O_2_ contents by 2.9, 1.9 and 8.4 folds, respectively, when compared to the normal group. This was coupled with a notable decrease in GSH content by 41% as compared to the normal group. Upon treatment with formoterol, oxidative stress was noticeably reduced, evidenced by the substantial decrements in MDA, NO, and H_2_O_2_ contents, contrary to a marked increase in GSH content (by 46.5%, 31%, 78% and 46%, respectively), (P < 0.0001, P < 0.0001, P < 0.0001, P < 0.01, respectively). Hence, formoterol effectively mitigated ketamine-induced oxidative stress. Administration of PKA inhibitor hindered formoterol–associated anti-oxidant effect with marked increment in MDA, NO, and H_2_O_2_ by 1.9, 1.5 and 4.2 folds, respectively, contrary to GSH reduced content by 40.4% (Fig. 4).

### The effect of formoterol on ketamine-injected mice's neuro-inflammation and apoptosis

**Fig. 5:**
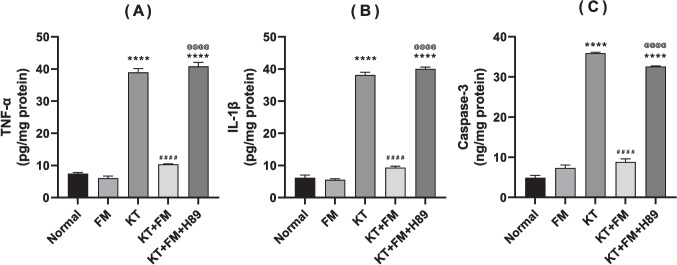
Effect of formoterol on ketamine-induced neuro-inflammation and apoptosis in bilateral hippocampal homogenates; (**A**) TNF-α, (**B**) IL-1β and (**C**) caspase-3. Values are expressed as mean ± SD; n = 6. ^****^P < 0.0001 vs normal group, ^####^P < 0.0001 vs ketamine (KT) group, ^@@@@^P < 0. 0001 vs treatment group (KT + FM)

Ketamine injection resulted in a significant elevation in the TNF-α and IL-1β besides the pro-apoptotic marker; caspase-3 by 5.4, 6.2, and 6.8 folds, respectively, as compared to the normal control. Treatment with formoterol mitigated ketamine-induced neuroinflammation and apoptosis with a notable decline in the formerly mentioned parameters by 75%, 77%, and 75%, respectively (P < 0.0001). Formoterol–induced anti-inflammatory and anti-apoptotic activities were abolished in mice which received the PKA inhibitor as the contents of the formerly mentioned inflammatory and apoptotic markers were increased by 3.8, 4.1, and 3.7 folds, respectively, in comparison with formoterol-treated group (Fig. 5).

### The effect of formoterol on ketamine-injected mice's PKA/cAMP/BDNF cascade

**Fig. 6:**
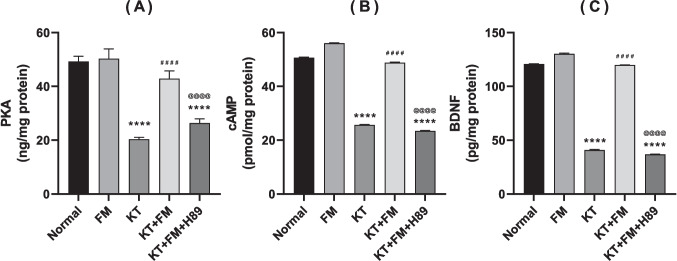
Effect of formoterol on ketamine-induced changes in bilateral hippocampal homogenates of PKA (**A**), cAMP (**B**), and BDNF (**C**) contents. Values are expressed as mean ± SD; n = 6. ^****^P < 0.0001 vs normal group, ^####^P < 0.0001 vs ketamine (KT) group, ^@@@@^P < 0. 0001 vs treatment group (KT + FM)

Ketamine group displayed a significant reduction in PKA, cAMP and BDNF contents by 59%, 50% and 67%, respectively. Formoterol-treated animals showed an obvious upsurge in PKA/cAMP/BDNF cascade reaching 2.1, 1.9 and 3 folds, respectively. PKA inhibitor administration eliminated formoterol modulatory effect on the formerly mentioned signaling pathway with prominent decline PKA, cAMP and BDNF contents by 38%, 52% and 69.5%, respectively, as compared to formoterol treatment group (Fig. 6).

### The effect of formoterol on ketamine-injected mice's neurotransmitter derangement

**Fig. 7:**
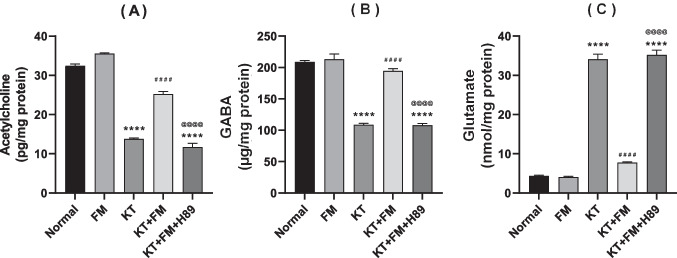
Effect of formoterol on ketamine-induced derangement in bilateral hippocampal homogenates of neurotransmitters: Ach (**A**), GABA (**B**), and glutamate (**C**) contents. Values are expressed as mean ± SD; n = 6. ^****^P < 0.0001 vs normal group, ^####^P < 0.0001 vs ketamine (KT) group, ^@@@@^P < 0. 0001 vs treatment group (KT + FM)

Ketamine injection reduced Ach and GABA contents by 58% and 49%, respectively, while it increased glutamate levels to reach eightfold as compared to normal mice. Formoterol reversed ketamine–induced neurotransmitter derangement by boosting Ach and GABA contents to 1.9 and 1.8 folds, respectively whereas the glutamate content was decreased by 77%. Mice which received the PKA inhibitor exhibited defective neurotransmitter signaling resembling that witnessed in ketamine group with obvious reduction in Ach and GABA contents by 54.9% and 44% contrary to increased glutamate content by 4.5 fold in comparison with formoterol-treated animals (Fig. 7).

### The effect of formoterol on ketamine-injected mice's synaptophysin content and PEA-3 gene expression

**Fig. 8:**
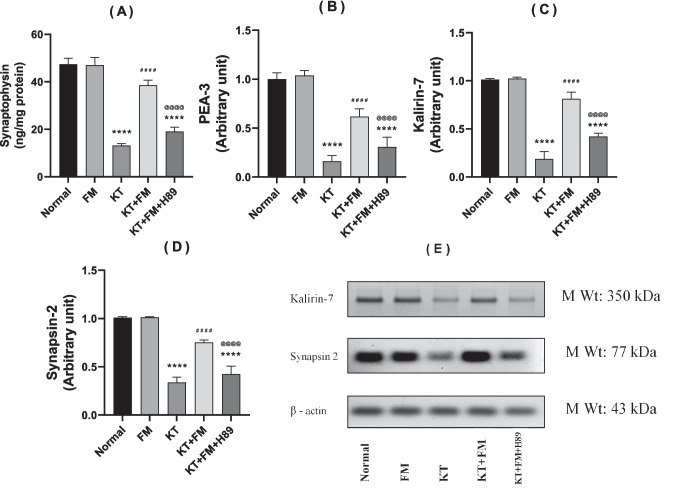
Effect of formoterol on ketamine-induced changes in hippocampal synaptic proteins: synaptophysin (**A**), PEA-3 (**B**); (n = 6) and Western blot analysis of right hippocampal homogenates for kalirin-7 (**C**), synapsin-2 (**D**) and (**E**); (n = 3). Values are expressed as mean ± SD. ^****^P < 0.0001 vs normal group, ^####^P < 0.0001 vs ketamine (KT) group, ^@@@@^P < 0. 0001 vs treatment group (KT + FM)

Ketamine group exhibited a significant decrement in synaptophysin content in addition to reduced gene expression of PEA-3 by 72% and 82%, respectively, as compared to the normal group. However, ketamine-injected mice treated with formoterol showed a notable increase in the formerly mentioned markers to 2.9 and 3.4 folds, respectively (P < 0.0001) indicating formoterol–induced synaptic growth and repair, an effect that was hindered by administration of the PKA inhibitor with prominent reduction in synaptophysin content and down regulation of PEA-3 by 51% and 49.2%, respectively in comparison with formoterol-treated group (Fig. 8: A & B).

### The effect of formoterol on ketamine-injected mice's kalirin-7 and synapsin-2 expression

In ketamine group, kalirin-7 and synapsin-2 were markedly downregulated by 81% and 67%, respectively, whereas administration of formoterol resulted in a marked upregulation of both markers by 4.3 and 2.3 folds, respectively. Formoterol enhanced synaptic plasticity was hindered upon administration of the PKA inhibitor with prominent downregulation of both synaptic markers by 47% and 44%, respectively in comparison with formoterol-treated animals (Fig. 8: C & D).

### The effect of formoterol on ketamine-injected mice's PSD-95 content and RIM-1α, GluN2A and GluN2Bgene expression

**Fig. 9:**
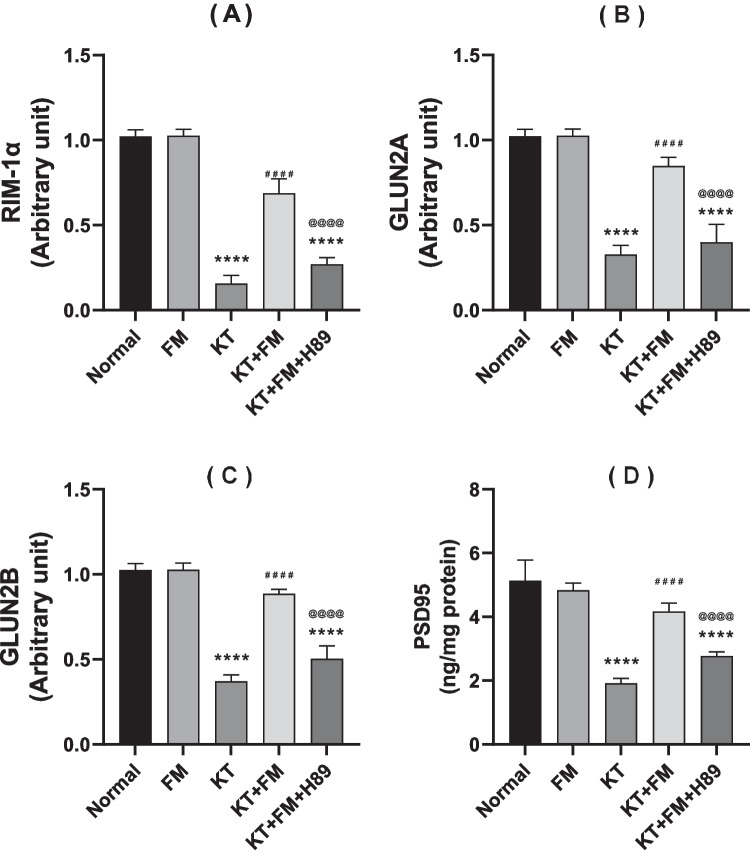
Effect of formoterol on ketamine-induced changes in hippocampal tissues (whole hippocampi and left hemispheres) for: RIM-1α (**A**), GluN2A (**B**), GluN2B (**C**) gene expression and bilateral hippocampal homogenates for PSD-95 content (**D**). Values are expressed as mean ± SD; n = 6. ^****^P < 0.0001 vs normal group, ^####^P < 0.0001 vs ketamine (KT) group, ^@@@@^P < 0. 0001 vs treatment group (KT + FM)

Ketamine group exhibited a significant reduction in RIM-1α, GluN2A, and GluN2B subunits gene expression besides PSD-95 reduced content by 86%, 68%, 63% and 62% respectively, in comparison with the normal group. In contrast, ketamine-injected mice treated with formoterol showed an upregulation of the gene expression of RIM-1α, GluN2A, and GluN2B subunits as well as an obvious increase in PSD-95 content reaching 4.7, 2.6, 2.3 and 2.2 folds, respectively (P < 0.0001). Formoterol enhanced glutamatergic transmission and augmented synaptic plasticity were abolished upon injection of the PKA inhibitor with marked decline in RIM-1α, GluN2A, and GluN2B subunits gene expression besides decreased PSD-95 content by 59.1%, 50.6%, 42%, and 33.2% as compared to formoterol–treated mice (Fig. 9).

### The effect of formoterol on ketamine-injected mice's brain histopathological alterations

**Fig. 10:**
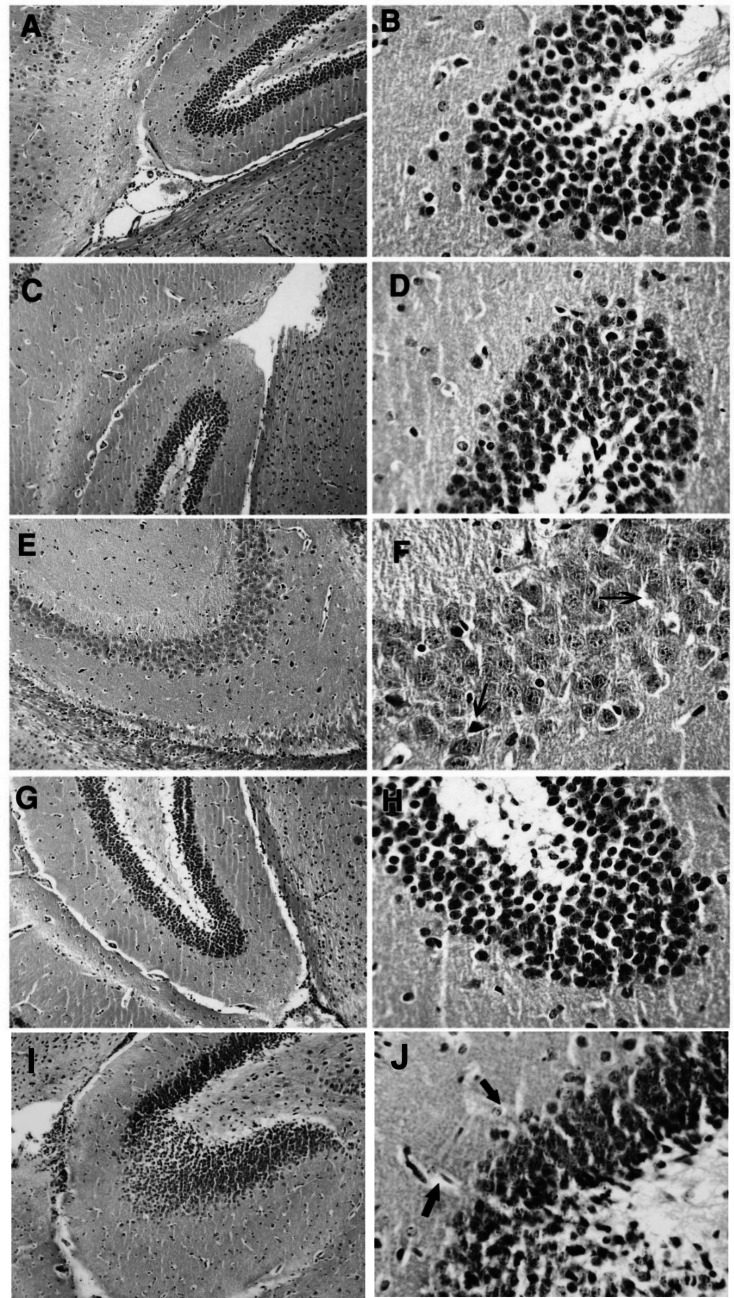
Effect of formoterol on ketamine-induced histopathological alterations in mice’s hippocampi using hematoxylin and eosin stain; A, C, E, G, I × 100 and B, D, F, H, J × 400. (**A**) & (**B**): normal group with normal histo-architecture showing normal arrangement of the pyramidal cells. (**C**) & (**D**): formoterol group (FM) with almost same picture as the normal group. (**E**) & (**F**): ketamine group (KT) showing loss of normal architecture of the hippocampus. Most of the pyramidal cells have pale nuclei and vacuolated cytoplasm (arrows). (**G**) & (**H**): KT + FM group photomicrographs showing a marked improvement with less obvious alteration in architecture. (**I**) & (**J**): PKA inhibitor group (KT + FM + H-89) with almost the same picture as KT group

**Fig. 11:**
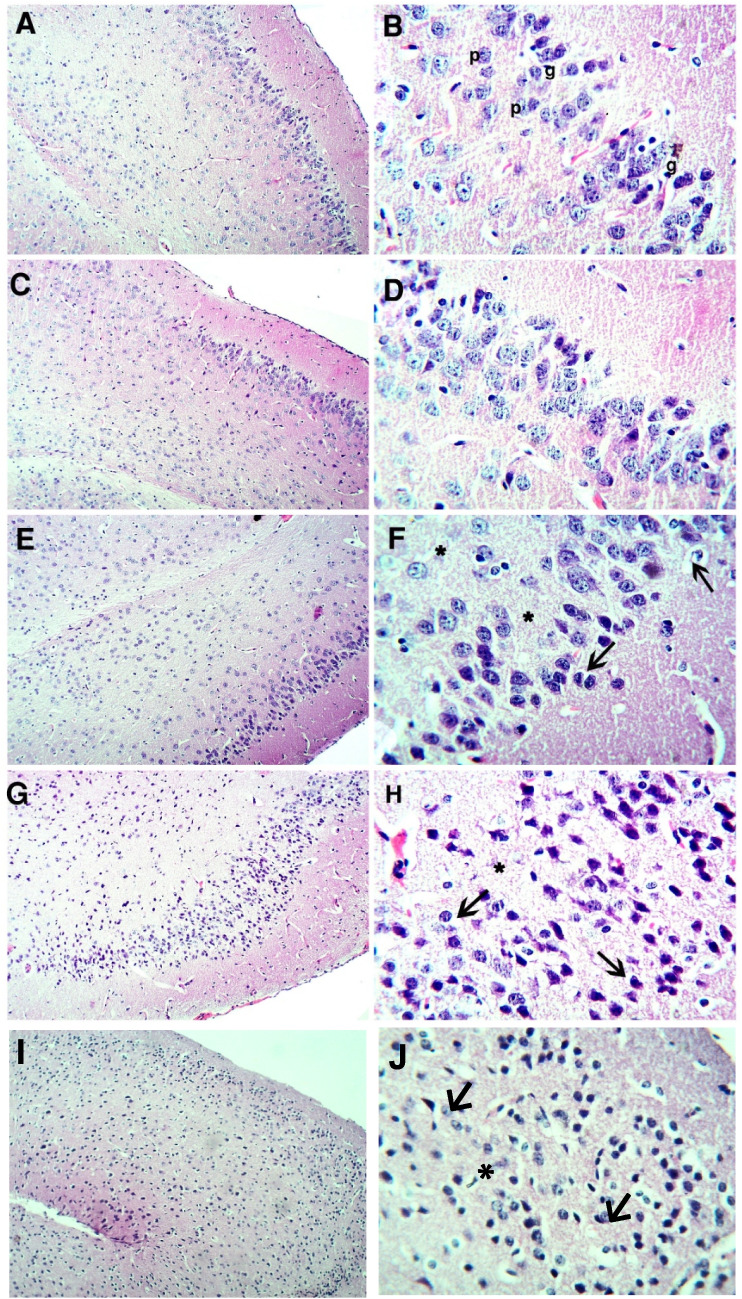
Effect of formoterol on ketamine-induced histopathological alterations in mice's prefrontal cortices using hematoxylin and eosin stain; A, C, E, G, I × 100 and B, D, F, H, J × 400. (**A**) & (**B**): normal group with normal histo-architecture showing normal arrangement of the pyramidal cells (p) and the glial cells (g). (**C**) & (**D**): formoterol group (FM) with almost same picture as the normal group. (**E**) & (**F**): ketamine group (KT) showing loss of normal cellular arrangement. The pyramidal cells in the majority appear with dark nuclei and vacculated cytoplasm with foamy appearance of the in-between neurons (asterisks). (**G**) & (**H**): KT + FM group photomicrographs showing a marked improvement with less obvious alteration in architecture. (**I**) & (**J**): PKA inhibitor group (KT + FM + H-89) with almost the same picture as KT group

Normal and formoterol-only groups exhibited no hippocampal and cortical histopathological alterations. In contrast, ketamine group demonstrated a disruption of the normal cellular arrangement. The pyramidal cells in the hippocampus and the prefrontal cortex displayed pale nuclei and vacuolated cytoplasm (arrows) with a foamy appearance of the inter-neuronal spaces in the prefrontal cortex (asterisks). Administration of formoterol to ketamine-injected mice resulted in a gradual improvement of their histopathological images, with less pronounced architectural alterations, indicating the protective effect of formoterol against ketamine-induced histopathological alterations in the hippocampus and prefrontal cortex. Administration of the PKA inhibitor hindered formoterol associated neuroprotection with similar histopathological alterations as those witnessed in the ketamine group in prefrontal cortical and hippocampal photomicrographs (Fig. 10 and 11).

## Discussion

This study aimed to evaluate the protective effects of formoterol on ketamine-induced schizophrenia-like behaviors in mice. Ketamine injection is widely recognized as a reliable model for studying schizophrenia, as it mimics various features including learning and memory deficits (Bitanihirwe and Woo 2011; Fond et al. 2020; Frohlich and Horn 2014). In this investigation, the ketamine group exhibited a significant deterioration in learning and memory functions, as evidenced by the marked reduction in the amount of time spent in the target quadrant and MEL in the MWM and the Y maze-spontaneous alteration behavior. These findings are consistent with previous studies indicating that ketamine administration was associated with a noticeable decline in learning and memory, both short- and long-term as demonstrated in the Y maze test and impaired performance in the Morris water maze on trainings and test days (Shi et al. 2021; Sun et al. 2011; Wang et al. 2006).

Ketamine injection has been reported to increase exploratory behavior in mice, indicating anxiety often associated with schizophrenia (Tapias-Espinosa et al. 2018). This effect was largely reflected on the exploratory activity which recorded an obvious increase in open field test without significant changes in latency, comparing to the normal group. Administration of formoterol to ketamine-injected mice resulted in significant improvements in learning and memory functions, as evidenced by increased time spent in target quadrant in the MWM test and the increase of spontaneous alteration behavior in the Y maze task. Formoterol administration led to a decrease in the anxiety elicited by ketamine, as indicated by the significantly decreased ambulation, rearing and grooming frequencies in the formoterol-treated groups compared to the ketamine group. Formoterol was also effective in improving ketamine induced impaired social interaction and anhedonic behavior indicating its ability to control most of schizophrenia-like behaviors.

The interplay between PKA, cAMP, and neuroplasticity involves intricate signaling pathways that are crucial for brain function and are frequently disrupted in schizophrenia. As a pivotal second messenger, cAMP significantly impacts neuroplasticity (Funk et al. 2012). In schizophrenia, abnormalities in cAMP-mediated signaling pathways, particularly in the frontal cortex, are well-documented and are hypothesized to underlie the cognitive impairments characteristic of the disorder (Braunewell et al. 2011). The cAMP signaling cascade is essential for regulating synaptic transmission and neuronal excitability—both fundamental to neuroplasticity. Disruptions in this pathway result in compromised synaptic function, contributing to the cognitive deficits observed in schizophrenia (Paspalas et al. 2013).

PKA, an enzyme activated by cAMP, plays a pivotal role in modulating cellular processes by phosphorylating various target proteins, including those critical for synaptic plasticity (Sahay et al. 2024). Altered PKA activity has been detected in the frontal cortex of individuals with schizophrenia, implicating its contribution to the disorder's underlying pathophysiology (Paspalas et al. 2013). Both cAMP and PKA regulate the expression of genes linked to synaptic function and plasticity, such as the BDNF via the transcription factor, CREB (Molteni et al. 2008).

BDNF exerts its influence on synaptic structure through interactions with proteins such as Rac1, where its activation in neurons is largely mediated by Kalirin (Yan et al. 2016). In neuropsychiatric conditions like schizophrenia, reduced Kalirin-7 expression significantly impairs BDNF-mediated processes essential for neurite outgrowth and dendritic branching (Yan et al. 2016). Beyond modulating synaptic activity, BDNF contributes to both pre- and postsynaptic modifications by regulating the synaptic proteome, balancing protein synthesis and degradation—processes vital for synaptic potentiation and structural plasticity (Edelmann et al. 2014). It regulates PSD-95 expression, which is critical for synaptic structure and function, and enhances microtubule dwell time in dendritic spines through TrkB receptor activation. Moreover, BDNF reverses synaptic density loss and restores mRNA and protein levels of synaptic plasticity-related proteins, including synaptophysin, in hippocampal neurons subjected to high glucose conditions (Zhong et al. 2019).

PKA is also capable of phosphorylating RIM1-α, that serves a crucial function in synaptic transmission and plasticity (Mäki-Marttunen et al. 2024). This phosphorylation may influence RIM1-α's functionality, which is critical for presynaptic plasticity and cognitive processes, both of which are commonly disrupted in schizophrenia (McGuire et al. 2017). Experimental studies using RIM1-α-deficient mice have demonstrated behaviors relevant to schizophrenia, including deficits in prepulse inhibition, altered responses to psychotomimetic agents, and impairments in social interactions (Hidalgo et al. 2021). These results indicate that dysregulation of RIM1-α function may contribute to the neurobiological mechanisms underlying schizophrenia-related cognitive and behavioral abnormalities (Kaeser et al. 2012).

Studies have established that postsynaptic RIM-1α is pivotal in facilitating the recycling of NMDARs, underscoring its importance for maintaining synaptic integrity and supporting the mechanisms underlying long-term memory formation. Experimental knockdown of RIM-1α was observed to result in a marked reduction in the surface localization of NMDARs. This phenomenon could potentially be attributed to enhanced internalization of NMDARs from the plasma membrane or a diminished rate of their reinsertion into the membrane, thereby affecting synaptic receptor dynamics and functional stability (Wang et al. 2018).

The reduction in synapsin-2 expression in various brain regions is linked to a downregulation of PEA-3 via cAMP in various psychotic disorders, including mood disorders and neurodegenerative disorders such as Alzheimer’s disease, owing to alterations in brain synaptic plasticity (Joshi et al. 2018). PEA-3, proteins belong to a subfamily of the E-twenty-six (ETS) domain superfamily of transcription factors, are expressed in the hippocampus (Kandemir et al. 2020). PEA-3 is crucial in regulating synaptic plasticity via promoting synapsin-2 gene expression (Petersohn et al. 1995). The current findings indicate that ketamine suppressed cAMP/PKA signaling, which likely resulted in the downregulation of the PEA-3 transcription factor and subsequent reduction in synapsin-2 levels. In addition, diminished BDNF and RIM-1α following suppressed PKA activity in mice received ketamine was associated with reduced kalirin‐7, PSD95, synaptophysin as well as GluN2A and GluN2B gene expression. The presynaptic deficits (involving impaired vesicle release due to altered synapsin-2 and RIM‐1α) combined with the postsynaptic deficits (due to disorganized receptor complexes from reduced PSD95 and dysfunctional GluN2B and kalirin‐7) may culminate in defective synaptic transmission and plasticity, affecting cognitive functions (An and Sun 2017; Sheng and Kim 2002). The present findings align with previous studies reporting alterations in the expression of GluN2A and GluN2B subunits in neuropsychiatric conditions including schizophrenia (Cardis et al. 2018; Liang and Zhang 2024). In the same context, schizophrenia is linked to a decreased expression of kalirin-7 and synapsin-2, that are linked to neurodegeneration and schizophrenia-like behaviors that impact learning and memory (LaRese et al. 2017; Mandela and Ma 2012). Similarly, a link between the reduction in PEA-3, RIM-1α and schizophrenia-related behaviors has been suggested previously (Blundell et al. 2010; Hidalgo et al. 2021).

In the current investigation, formoterol mitigated these effects by enhancing cAMP/PKA activity, thereby upregulating BDNF, PEA-3 and RIM-1α expression. Accordingly, it significantly boosted the expression of kalirin-7, synapsin 2, GluN2A, GluN2B, PSD95, and synaptophysin, indicating an antipsychotic effect via attenuation of synaptic disturbances. This demonstrates a clear mechanistic interplay between β2-adrenergic signaling and synaptic plasticity proteins, mediated through the cAMP/PKA/BDNF-PEA-3 and RIM-1α signaling, which underscores the protective effects of formoterol. This finding was further advocated by the present results from the PKA-inhibitor group that showed no notable difference compared to the ketamine-only group, irrespective of formoterol treatment.

The reduction in BDNF contributes to GABAergic deficits manifesting schizophrenia-related symptoms, including altered synaptic responses and cognitive deficits (Fujihara et al. 2020; Hashimoto et al. 2005). In alignment with previous reports, the marked drop in BDNF levels in ketamine group was accompanied by diminished GABA levels and notable cognitive impairments (Li et al. 2020; Wan et al. 2024). Treatment with formoterol counteracted these effects by substantially elevating BDNF levels, thereby restoring them toward baseline values, and enhancing GABAergic tone, as evidenced by elevated brain GABA levels. The reestablishment of the BDNF–GABA axis is likely to have facilitated the observed enhancements in cognitive flexibility and synaptic functionality in mice treated with formoterol.

Several studies have linked ketamine with oxidative stress (Bove et al. 2020; Liu et al. 2015; Mert et al. 2019). In schizophrenia, NMDA receptor hypofunction is associated with increased oxidative stress, which further exacerbates synaptic dysfunction (Foster et al. 2017). Redox imbalance can directly affect synaptic function by altering antioxidant defenses like the glutathione system (Baxter et al. 2015) which has been linked to decreased BDNF (Harb et al. 2021; Lech et al. 2021).

This study corroborated these findings by showing increased oxidative stress in the ketamine group, evidenced by significant elevations in MDA, NO and H_2_O_2_ levels, and a marked reduction in GSH level. Formoterol administration significantly mitigated ketamine-induced oxidative stress by reducing MDA, NO and H_2_O_2_ and increasing GSH brain levels.

Oxidative stress also influences the pro-inflammatory cytokines production, leading to neuro-inflammation linked to cognitive deterioration in schizophrenia patients (Barron et al. 2017). Cholinergic dysfunction and cognitive deterioration in schizophrenia have been linked to elevated levels of TNF-α and IL-1β (Erbağci et al. 2001).

Beyond the part oxidative stress plays in synaptic dysfunction, neuroinflammation can also disrupt synaptic function and plasticity. Elevated levels IL-1β and TNF-α could impair plasticity and contribute to schizophrenia-like behavioral (Wei et al. 2022).

In addition to the excessive glutamate release mediated by the inhibitory effect of ketamine on GABAergic neurons (Duarte et al. 2012; Lander et al. 2019; Yang et al. 2017). TNF-α has been implicated in promoting the release of glutamate by activated microglia (Chen et al. 2012). Furthermore, TNF-α has been shown to facilitate the activation of caspase-3, which suppresses glutamate uptake mechanisms, thereby exacerbating excitotoxicity (Jarskog et al. 2007; Olmos and Lladó, 2014) which further disrupts synaptic function. In this context, essential synaptic markers like synaptophysin are lost as a result of neuroinflammation and excitotoxic exposure (Rao et al. 2012).

Accordingly, ketamine-induced increase in TNF-α levels has been linked to excitotoxicity, and neuroinflammation-associated synaptic dysfunction, and apoptosis. This relationship was demonstrated in this study, wherein ketamine-injected mice exhibited significant elevations in TNF-α, IL-1β, glutamate and caspase-3 activity along with disrupted synaptic markers. Notably, treatment with formoterol led to a substantial improvement in these parameters, suggesting its potential to alleviate ketamine-induced synaptic failure, neuroinflammation, excitotoxicity and apoptosis.

The improvement in schizophrenia-like behaviors, as demonstrated in behavioral assessments following treatment with formoterol, may be attributed to its ability to ameliorate synaptic deficits by mitigating oxidative stress and inflammation in the brain (Khalili et al. 2018; Muhammad et al. 2019; Wei et al. 2020). Consistent with these findings, previous studies have emphasized the antioxidant and anti-inflammatory properties of formoterol, which have been demonstrated across various animal models of central nervous system disorders (Damo et al. 2023; Khidr et al. 2023; Salazar-Degracia et al. 2017).

The interplay between oxidative stress, neuroinflammation—associated with ketamine administration—and the cAMP/PKA signaling pathway is complex, with these mechanisms being closely interconnected. Notably, oxidative stress can diminish intracellular cAMP levels by enhancing the activity of NADPH oxidase, a key producer of reactive oxygen species (Cuello et al. 2021; Saha et al. 2011). Furthermore, both cAMP and PKA are redox-sensitive, as oxidative stress may induce oxidative modifications of proteins, leading to functional impairments. It's worth noting that PKA has been implicated in the attenuation of neuroinflammation through multiple mechanisms. These include its antioxidant properties, which mitigate oxidative stress, and its ability to reduce excitotoxicity by modulating glutamate signaling pathways (Dai et al. 2010; Uematsu et al. 2015; Zhang et al. 2019). Additionally, PKA may exert anti-inflammatory effects by influencing the polarization of microglia towards an anti-inflammatory phenotype (Hu et al. 2021). Furthermore, PKA can regulate the release of cytokines from astrocytes, thereby contributing to the modulation of the neuroinflammatory milieu (Li-Yan et al. 2021). This was confirmed in our study, as the PKA-inhibitor group exhibited persistent exacerbation of oxidative stress, neuroinflammation, and other related biomarkers, even in the presence of formoterol administration. This highlights the pivotal role of PKA in formoterol underlying mechanistic pathway.

Meanwhile, formoterol was primarily developed as a bronchodilator for asthma and COPD, the dose employed in our psychiatric study corresponds to supratherapeutic ranges relative to pulmonary use. Such supratherapeutic dosing is a common preclinical strategy to ensure CNS exposure (Kumar and Talwar 2025). Recent work on BBB pharmacology confirms that small molecules such as β2 adrenergic agonists can achieve measurable CNS penetration, supporting the plausibility of neuropsychiatric effects (Kumar and Talwar 2025). Indeed, systemic formoterol has been shown to alter endocannabinoid tone in the periaqueductal gray (Peterson et al. 2024), promote mitochondrial biogenesis and cognitive recovery after traumatic brain injury (Vekaria et al. 2020), and ameliorate neuroinflammation in murine models (Abdel Rasheed et al. 2018), providing convergent evidence of central receptor engagement.

Chronic β2 adrenergic agonism was linked to potential cardiovascular risks, including tachycardia, as well as the possibility of receptor desensitization or down regulation with sustained administration (Hanania et al. 2019). Because schizophrenia requires long term pharmacotherapy, the potential for tolerance in the CNS warrants careful consideration. However, available preclinical data suggest that formoterol retains efficacy in the brain after repeated dosing, and long term clinical studies in COPD patients have demonstrated that therapeutic doses are safe and well tolerated, with adverse effects mainly limited to mild cardiovascular symptoms (Hanania et al. 2019).Importantly, the adverse effect profile of formoterol is considerably milder than that of conventional antipsychotics. Long acting β2 agonists are primarily associated with transient cardiovascular effects and mild tremor, but they do not carry the substantial metabolic, extrapyramidal, and endocrine side effects characteristic of chronic antipsychotic therapy. For example, antipsychotics are strongly linked to weight gain, insulin resistance, dyslipidemia, and tardive dyskinesia, which contribute to the elevated cardiovascular and metabolic burden in schizophrenia patients (Pillinger et al. 2020). In contrast, formoterol has been safely used for years in asthma and COPD management, with a well characterized and relatively benign safety profile (Hanania et al. 2019).

Taken together, these observations suggest that β2 adrenergic modulation represents a distinct and complementary therapeutic approach. By enhancing synaptic plasticity and attenuating neuroinflammation, formoterol may provide benefits beyond dopaminergic and serotonergic blockade, with the potential for improved tolerability in long term schizophrenia treatment.

## Conclusion

Based on these findings, the present study proposes that formoterol exerts a protective role against schizophrenia-like behaviors by hampering neuroinflammation and oxidative stress and restoring synaptic plasticity. These effect could be partially ascribed to its ability to modulate cAMP/PKA/BDNF-PEA-3 and RIM-1α signaling. We recommend further research to evaluate its efficacy in humans and its potential as a therapeutic approach for schizophrenia management.

## Study limitations and future directions

1. Ketamine does not fully recapitulate the chronic and neurodevelopmental features of schizophrenia (for example: genetic models should be considered).
2. Other mechanisms other than redox imbalance & inflammation might affect synaptic function (including for example endogenous neurosteroid regulation, mitochondrial dynamics, and microglial polarization) that could be tackled in future studies.
3. Other signaling cascades downstream of β2 receptor (like Erk1/2) might be contributing to formoterol’s beneficial effects (more studies are warranted).
4. In this study, male mice were selected to reduce hormonal variability and to align with prior ketamine-based models that have established robust behavioral and molecular phenotypes in males. While this approach enhances internal consistency, we recognize that schizophrenia affects individuals of all sexes. Future studies could incorporate both male and female cohorts to explore potential sex-specific responses to β2-adrenoceptor modulation and to improve translational relevance.
5. Although formoterol is recognized as a highly selective β2‑adrenergic receptor agonist, potential off‑target effects cannot be fully excluded. Likewise, H‑89, while widely used as a PKA inhibitor, may also inhibit other kinases. Thus, while our findings strongly implicate β2AR‑cAMP/PKA signaling in mediating the protective effects of formoterol, the involvement of other culprits. Warrants further research.
6. Chronic administration of formoterol should be addressed to investigate the possibility of receptor desensitization and tolerance.

## Supplementary Information

Below is the link to the electronic supplementary material.

Supplementary file1 (DOCX 743 KB)

## Data Availability

Experimental data are available from the corresponding author upon reasonable request.

## Abbreviations

Ach: Acetylcholine
FM: Formoterol
KT: Ketamine
PKA Inhb: Protein kinase A inhibitor
β2AR: β2-Adrenergic receptor
MEL: Mean escape latency
RIM-1α: Rab3-interacting molecule- 1α
