# Supplementary figures and images for: Targeting beta-2 adrenergic receptor attenuates schizophrenia-like behavioral effects induced by ketamine in mice: cAMP/PKA/BDNF-PEA-3 and RIM-1α signaling pathways involvement
Source: Saudi Pharm J. 2026 Apr 29;34(2):24. doi: 10.1007/s44446-026-00079-x (PMC13129144; doi:10.1007/s44446-026-00079-x)

**Western (uncropped)**

beta actin


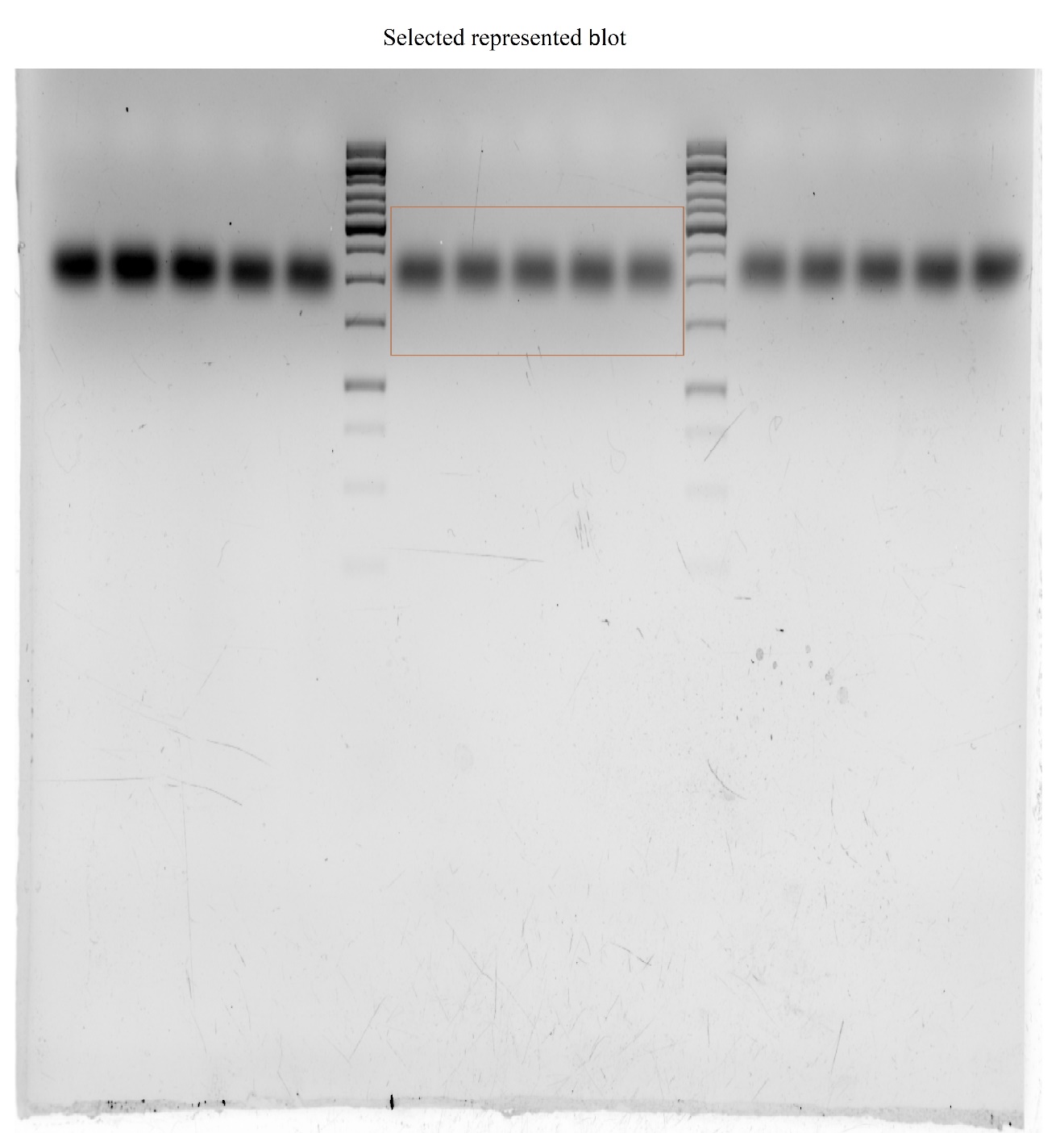


KALIRIN7


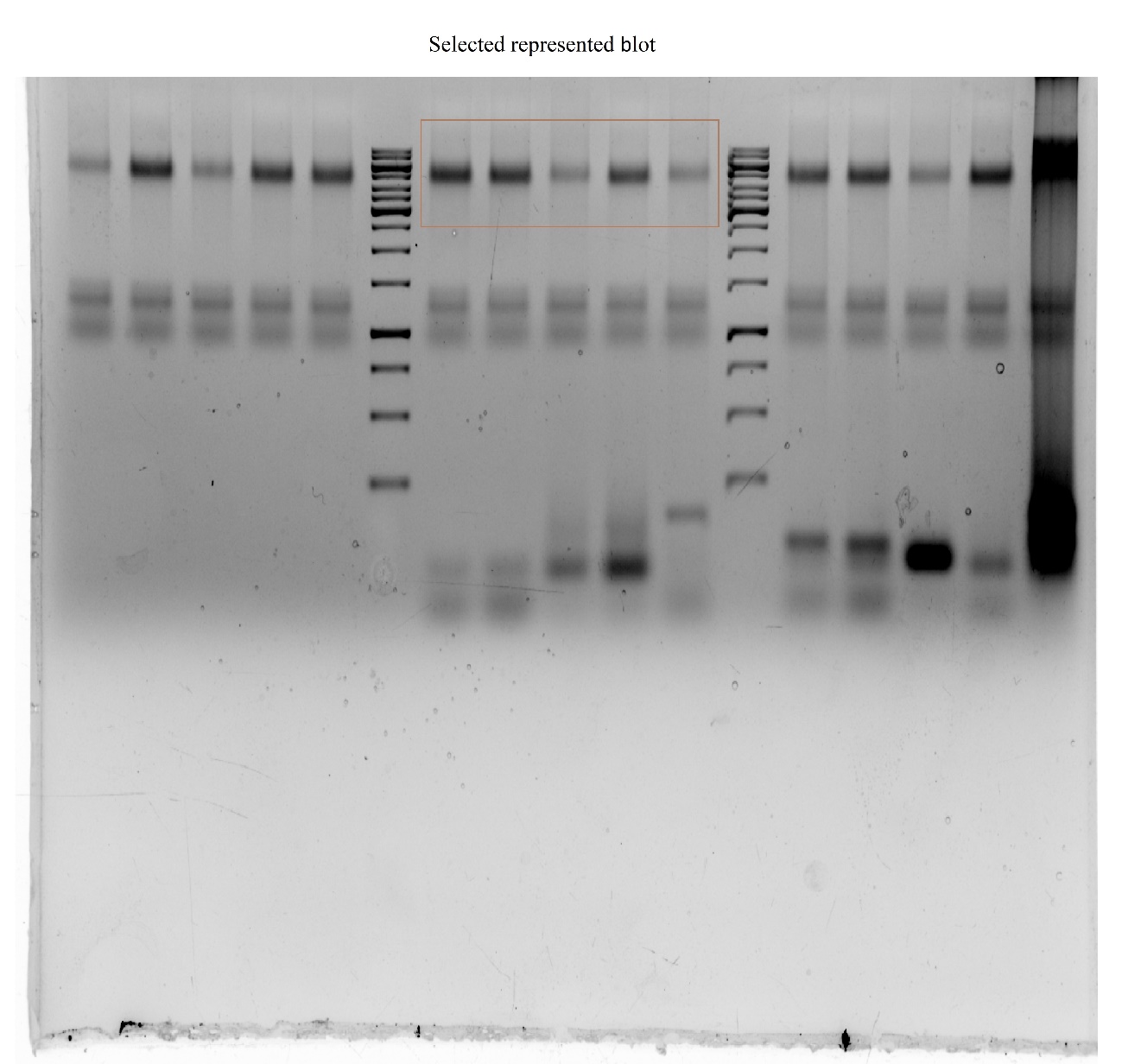


Synapsin 2


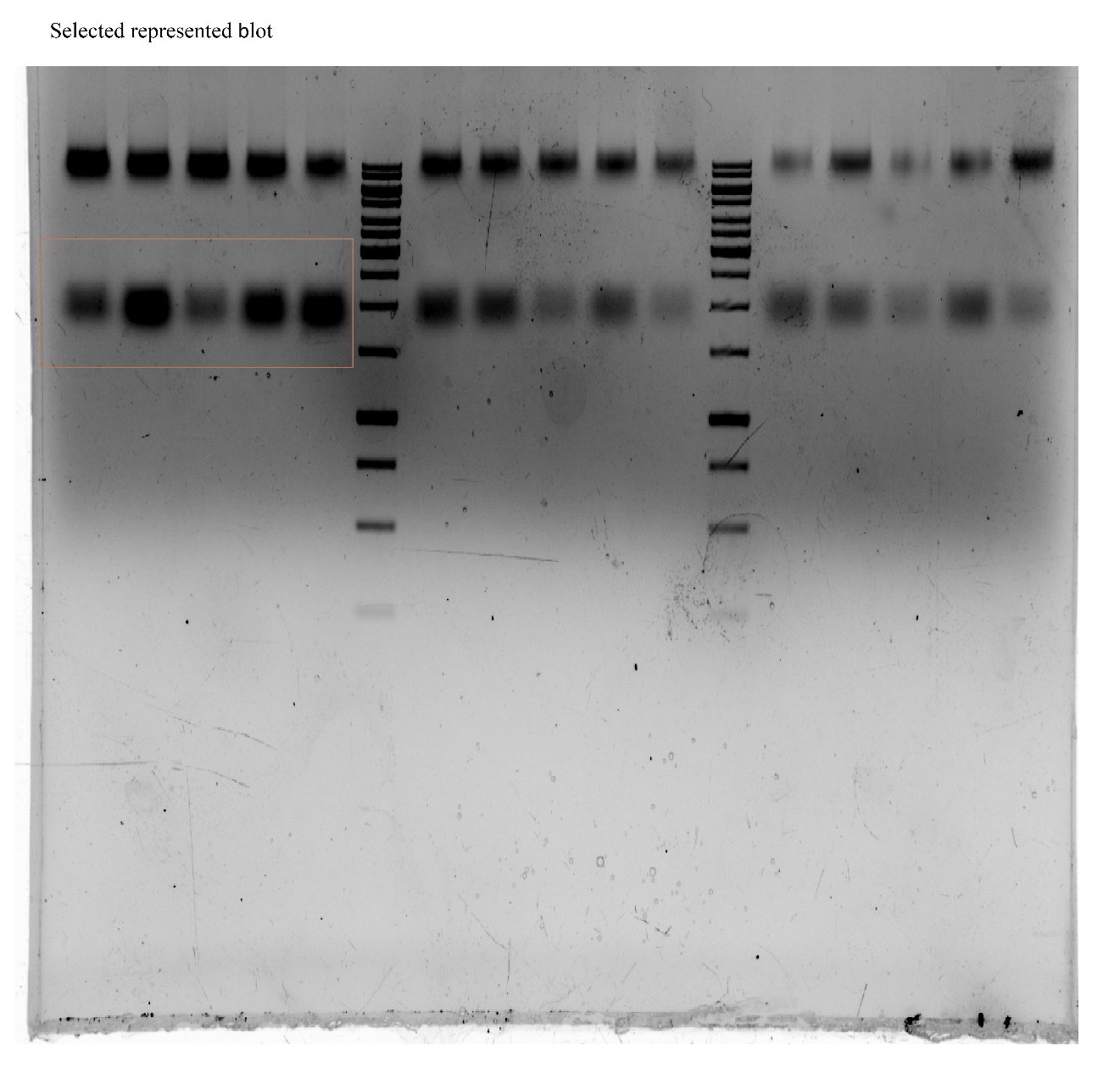

Supplement: Supplementary file 1 — Supplementary file1 (DOCX 743 KB) [file 44446_2026_79_MOESM1_ESM.docx]
